# Postprandial Increase in Blood Plasma Levels of Tissue Factor–Bearing (and Other) Microvesicles Measured by Flow Cytometry: Fact or Artifact?

**DOI:** 10.1055/s-0038-1642021

**Published:** 2018-04-16

**Authors:** Morten Mørk, Morten H. Nielsen, Rikke Bæk, Malene M. Jørgensen, Shona Pedersen, Søren R. Kristensen

**Affiliations:** 1 Department of Clinical Biochemistry, Aalborg University Hospital, Aalborg, Denmark; 2 Aalborg AF Study Group, Aalborg University Hospital, Aalborg, Denmark; 3 EVsearch.dk, Aalborg, Denmark; 4 Department of Clinical Immunology, Aalborg University Hospital, Aalborg, Denmark; 5 Department of Clinical Medicine, Aalborg University, Aalborg, Denmark

**Keywords:** extracellular vesicles, tissue factor, phosphatidylserine, lipoproteins, flow cytometry

## Abstract

Tissue factor (TF)–bearing microvesicles (MVs) and exosomes may play a role in hemostasis and thrombosis. MVs may be quantified by flow cytometry (FC)–based detection of phosphatidylserine (PS)-positive submicron particles carrying specific antigens, although interference from lipoproteins complicates this approach. In this study, we evaluated the effect of food intake on blood levels of TF-bearing particles measured by FC and small extracellular vesicles (EVs) measured by a protein microarray–based test termed EV Array. Platelet-free plasma (PFP) was obtained from 20 healthy persons in the fasting state and 75 minutes after consumption of a meal. Postprandial changes in the concentration of PS-positive particles, including subgroups binding labeled antibodies against TF, CD41, CD146, and CD62E, respectively (FC), small EVs (EV Array), and TF antigen and procoagulant phospholipids (PPLs) were measured. Furthermore, we tested the effect on FC results of in vitro addition of lipoproteins to fasting PFP. We found significantly increased plasma concentrations of PS-positive particles and all examined subgroups postprandially, while no changes in small EVs, PPL, or TF antigen levels were found. Levels of all types of particles measured by FC were also elevated by lipoprotein spiking. In conclusion, meal consumption as well as in vitro addition of lipoproteins to fasting plasma induces increased levels of PS-positive particles as measured by FC, including TF-positive subtypes and subtypes exposing other antigens. While the observed postprandial increase may to some extent reflect elevated MV levels, our results indicate a substantial interference from lipoproteins.

## Introduction

Tissue factor (TF) has been established as a pivotal element in activation of the clotting cascade. 1 It is well described that TF expressed subendothelially in blood vessels comes into contact with blood upon tissue injury and, in combination with activated FVII, initiates the coagulation process. 2 However, TF is also produced in monocytes and may furthermore be expressed in platelets and activated endothelial cells. 1 In plasma, TF circulates both in its full-length form incorporated in the membranes of circulating cell–derived extracellular vesicles (EVs) and in an alternatively spliced soluble form. 3 4 5 The role of blood-borne TF in the coagulation process is not clear, 2 5 but TF-bearing EVs (TF+ EVs) have been suggested to play a role in hemostasis and thrombus formation, 6 7 and TF activity and TF+ EVs quantitated by flow cytometry (FC) have been shown to be increased in various thrombogenic conditions. 1

Although consensus on the nomenclature of different types of EVs has not been achieved, 8 smaller sized EVs, typically with a diameter of less than 150 nm, may be termed exosomes, whereas larger EVs are often referred to as microvesicles (MVs). 9 While these two types of EVs are formed and released from cells by different mechanisms, 10 11 indications exist that both types possess the ability to carry TF in their phospholipid bilayer membrane. 1 5

A significant postprandial increase in plasma concentration of particles within the size range of exosomes and MVs has been demonstrated using nanoparticle tracking analysis 12 and tunable resistive pulse sensing (TRPS). 12 13 However, these methods lack the ability to distinguish between EVs and other submicron particles, including phospholipid monolayer–bounded lipoproteins such as chylomicrons, low-density lipoproteins (LDLs), and very low-density lipoproteins (VLDLs), which are present in plasma and display an altered relative distribution postprandially. 13 14 FC enables some characterization of the particles by fluorescence-based detection of phosphatidylserine (PS) combined with cell-type specific antigens and it has previously been reported in FC-based studies that blood concentrations of MVs are increased in the postprandial state. 15 16 17 However, recent investigations have indicated that MV measurement by FC on plasma may suffer from considerable interference from lipoproteins, which are not eliminated by state-of-the-art MV isolation methods. 13 Although PS is a well-established marker of MVs 10 18 19 and has been used for MV labeling in FC-based investigations, 13 16 the sensitivity 18 as well as the specificity 20 of PS-positive (PS+) particles as indicators of MVs can be questioned.

EV Array is a protein microarray–based technique designed for measurement of small EVs. 21 Biotinylated antibodies against CD9, CD63, and CD81, often regarded as exosome markers, but actually also present in MVs, 22 can be used in EV Array for sensitive detection of small EVs. 21

The aim of this study was to investigate the impact of food ingestion on plasma levels of PS+ particles (using FC) and small EVs (using EV Array) in general and their TF-bearing subtypes in particular as well as the effect of prandial state on levels of circulating TF antigen (Ag), TF activity, and procoagulant phospholipids (PPLs). Considering the recent questioning of the reliability of FC-based MV measurements, 13 we evaluated the impact of prandial state and, parallelly, the impact of in vitro addition of lipoproteins to fasting plasma on levels of particles positive and negative for PS and other markers indicating cell origin, discussing the potential influence of lipoproteins on postprandial changes of these analytes and the resulting limitations of FC in MV measurements on plasma.

## Materials and Methods

### Study Population and Sample Handling

In this study, which was approved by the local research ethics committee, we analyzed platelet-free plasma (PFP) samples from 20 volunteers (13 females and 7 males) with a median age of 44 (interquartile range [IQR], 33–58) years who were considered healthy based on a health status questionnaire. Additional blood tests were performed to screen for ongoing disease and to measure total cholesterol and triglyceride (TG) concentrations in the fasting and postprandial samples. From each person, a fasting venous blood sample was collected in a 9-mL Vacuette 3.2% sodium citrate plastic tube (Greiner Bio-One, Kremsmünster, Austria) in the morning before consumption of a nonstandardized breakfast with a choice of bread, butter, cheese, jam, marmalade, cake, coffee, tea, milk, and sugar. Seventy-five minutes following commencement of breakfast, a postprandial sample was collected. The first 3.5 mL of blood from each sampling were discarded. Samples were centrifuged twice at 2,500 g for 15 minutes at room temperature, as recommended by Lacroix et al, 23 to obtain PFP, which was stored at −80°C until analysis.

### Flow Cytometry

Plasma levels of MVs were evaluated by a modified FC approach based on a previously described method, 24 using a BD FACSAria III High Speed Cell Sorter (BD Biosciences, San Jose, California, United States). For each analysis, 50 μL of freshly thawed PFP were transferred to a TruCount tube (BD Biosciences) containing a lyophilized pellet, releasing a known number of fluorescent beads as an internal standard and enabling particle concentration determination according to the insert instruction of the manufacturer. Subsequently, 7 µL of fluorescein isothiocyanate (FITC)–conjugated lactadherin (Haematologic Technologies Inc., Essex Junction, Vermont, United States) were added to each sample, utilizing the binding affinity of lactadherin for PS, 25 to label particles exposing PS. All particles within the defined size gate (see below) were collectively designated as particles. Particles positively stained with FITC-conjugated lactadherin (which binds to PS) were designated as PS+ particles. To identify PS+ particles originating from platelets, 26 particles were further labeled with 3 µL of allophycocyanin-conjugated antihuman CD41 IgG1, κ (BioLegend, San Diego, California, United States). We identified endothelium-derived particles 27 by labeling with 20 µL of phycoerythrin (PE)-conjugated antihuman CD146 IgG1, κ (BD Biosciences), and furthermore activated endothelium-derived particles 27 by labeling with 20 µL of PE-conjugated antihuman CD62E IgG1, κ (BD Biosciences). TF-bearing (TF+) particles were identified by labeling with 20 µL of PE-conjugated antihuman TF IgG1, κ (BioLegend). TF+ particles that were not positively stained with FITC-conjugated lactadherin were designated TF+ PS− particles. Isotype controls matching each antibody were used as negative controls. After 30 minutes of incubation at 4°C in the dark, 200 µL of Dulbecco's phosphate-buffered saline (PBS) buffer (Lonza, Basel, Switzerland) that had been filtered through a sterile 0.2-µm Q-Max syringe filter (Frisenette, Knebel, Denmark) were added to each labeled sample. A size gate was established by preliminary standardization experiments using a blend of size-calibrated fluorescent polystyrene beads with sizes ranging from 0.2 to 0.9 μm. The upper and outer limits of the gate were established just above the size distribution of the 0.9-µm beads and the lower limit was set to include the 0.2-µm beads to obtain a size gate of about 0.2–1.0 µm in a forward (FSC) and side scatter (SSC) setting. A discriminator was set to 200 at the SSC-H parameter (instead of FSC-H) to avoid exclusion of smaller sized particles. Event numbers were counted at a maximal flow rate of 20,000 events/second and stopped after 60 seconds or when the MV gate reached at least 500,000 events. Flow rates of postprandial samples with increased particle numbers were adjusted to comply with the maximal flow rate limit.

To study the effect of in vitro addition of lipoproteins to plasma, four fasting PFP samples were spiked with an LDL isolate and a VLDL isolate, respectively, prior to the same labeling and incubation steps and FC analysis as described above. However, it was performed at a later time point on different plasmas and different batches of antibodies. One part of human LDL 5 mg/mL (Kalen Biomedical, Montgomery Village, Maryland, United States) was added to 10 parts of PFP and, parallelly, 1 part of human VLDL 1 mg/mL (Kalen Biomedical) was added to another 10 parts of PFP, resulting in increases in TG concentration of 0.1 and 0.2 mmol/L, and increases in cholesterol concentration of 2.2 and 0.2 mmol/L, respectively. As controls, we tested the effect of addition of the LDL and VLDL isolates and performing the incubation steps on samples not containing PFP, which was, in these control samples, replaced by an equal amount of PBS.

### EV Array

For small EV analysis, EV Array, allowing for quantification and phenotyping of this EV subtype, was applied. Microarray slides were printed as described by Jørgensen et al, 28 and the following antibodies or proteins were printed: anti-CD63, anti-CD41 (Biolegend), anti-CD9, anti-CD81 (LifeSpan BioSciences, Inc., Seattle, Washington, United States), antihuman TF (R&D Systems Inc., Minneapolis, Minnesota, United States), anti-CD146 (P1H12, Abcam, Massachusetts, United States), anti-CD62E (Thermo Scientific, Waltham, Massachusetts, United States), and lactadherin (Haematologic Technologies). All antibodies and proteins were printed in triplicates at 200 µg/mL diluted in PBS containing 5% glycerol.

Catching and visualization of the EVs were performed as described previously by Jørgensen et al 29 with the following modifications: After blocking, 10 µL of freshly thawed PFP diluted in wash buffer (0.05% Tween 20 [Sigma-Aldrich, St. Louis, Missouri, United States] in PBS) were incubated at room temperature for 2 hours followed by an overnight incubation at 4°C. Following a wash, the slides were incubated for 2 hours with detection antibodies. The detection was performed with an “exosome cocktail,” which is a combination of fluorescently labeled versions of the exosome-related markers anti-CD9, anti-CD63, and anti-CD81 (Ancell Corporation, Stillwater, Minnesota, United States) diluted 1:1,500 in wash buffer. After a wash, a subsequent 30-minute incubation step with cyanine 5 (Cy5)–labeled streptavidin (1:1,500; Life Technologies, Carlsbad, California, United States) in wash buffer was performed for detection. Prior to scanning at 635 nm, the slides were washed once in wash buffer and once in MilliQ water and dried using a Microarray High-Speed Centrifuge (ArrayIt, Sunnyvale, California, United States). Scanning and spot detection was performed as previously described. 29

### Additional Analyses

PPL-dependent clotting time (PPL-CT) was measured with STA Procoag PPL (Diagnostica Stago, Asnières, France) on an STA Compact Coagulation Analyzer (Diagnostica Stago).

TF Ag was measured by Imubind antihuman Tissue Factor ELISA (Sekisui Diagnostics, Lexington, Massachusetts, United States) using a SpectraMax M2 Microplate Reader (Molecular Devices, Sunnyvale, California, United States) measuring the absorbance at a wavelength of 450 nm. In the following, we refer to this method as TF Ag _ELISA_ .

A supplemental method for TF Ag measurement, represented by a modified version of EV Array with polyclonal anti-TF antibodies (R&D Systems Inc.) as capturing agents and biotin labeled polyclonal anti-TF antibodies (R&D Systems Inc.) as detecting agents, was applied and designated TF Ag _EV Array_ .

TF activity was evaluated with Tissue Factor Human Chromogenic Activity Assay Kit (Abcam, Cambridge, UK), and the absorbance at a wavelength of 405 nm was measured with SpectraMax M2 Microplate Reader (Molecular Devices).

Lipid levels were measured as routine analyses at the Department of Clinical Biochemistry, Aalborg University Hospital, using a Cobas 8000 Modular Analyzer (Roche Applied Science, Penzberg, Germany).

### Statistics

Statistical analyses were performed and graphs created using GraphPad Prism, version 6.01 (GraphPad Software, Inc., La Jolla, California, United States). Data, for which a normal distribution was not rejected when applying the Anderson–Darling test, were presented with mean value and standard deviation (SD), while *p* -value for pairwise comparison was calculated using paired *t* -test and degree of correlation indicated by Pearson's *r* . Data, for which a normal distribution was rejected, were presented with median value and IQR, while *p* -value for pairwise comparison was evaluated using Wilcoxon's matched pairs signed-rank test and degree of correlation indicated by Spearman's *r _s_* .

Two-sided *p* -values were given, and values below 0.05 considered significant.

Pooled coefficient of variation (CV) indicating the degree of consistency of results was calculated as the square root of the mean of the squared CVs for measurements on each subject.

## Results

### Flow Cytometry

**Fig. 1:**
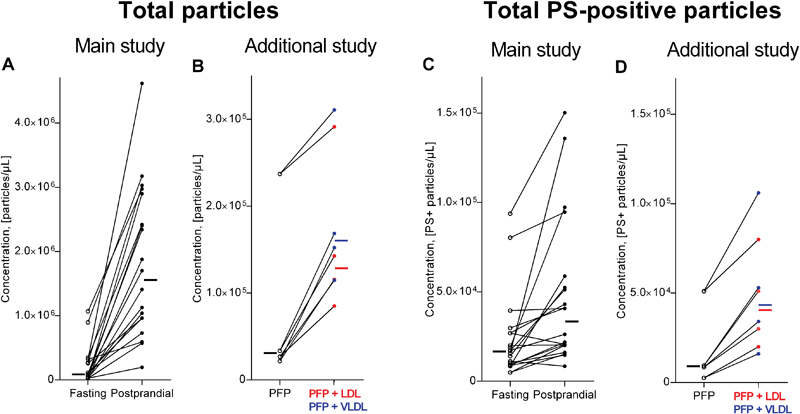
Fasting and postprandial concentration of ( **A** ) total particles and ( **C** ) phosphatidylserine-positive (PS+) particles measured by flow cytometry and, for comparison, fasting plasma before and after addition of VLDL or LDL ( **B and D** ). Lines combine fasting levels ( *open circles* ) with postprandial levels ( *closed circles* ) for each study participant, and for the additional study the levels before ( *open circles* ) and after addition of VLDL ( *blue closed circles* ) and LDL ( *red closed circles* ). Small bars to the left of the fasting and to the right of the postprandial results (or after addition of VLDL [ *blue* ] or LDL [ *red* ]) indicate the median of all results.

**Fig. 2:**
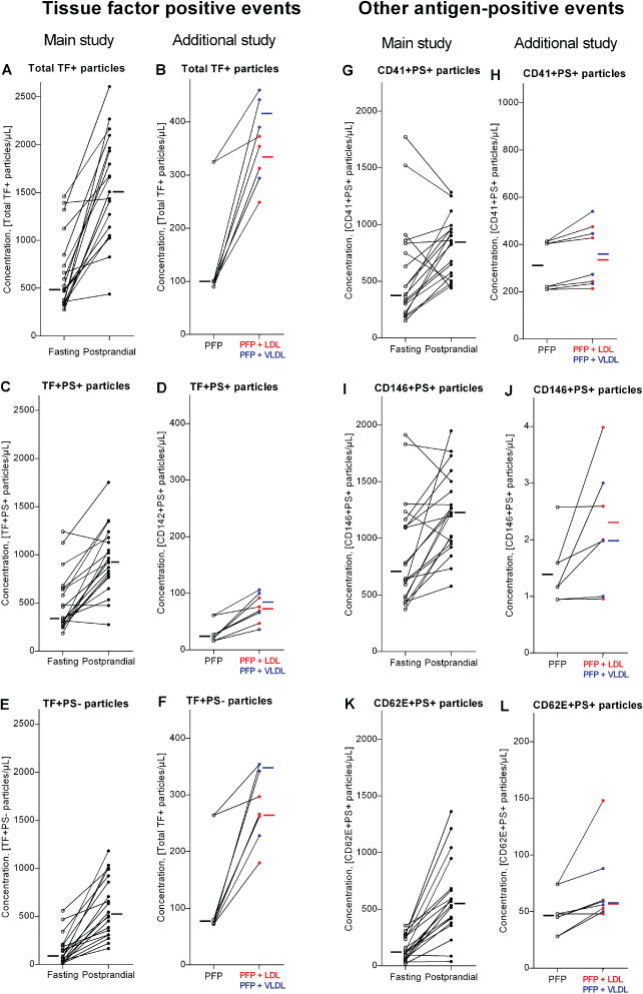
Fasting and postprandial concentration of ( **A** ) total tissue factor-positive (TF+) particles, ( **C** ) TF+ phosphatidylserine-positive(PS+) particles, ( **E** ) TF+ phosphatidylserine-negative (PS−) particles, ( **G** ) CD41+ PS+ particles, ( **I** ) CD146+ PS+ particles, and ( **K** ) CD62E+ PS+ particles measured by flow cytometry. The results for the same particle subtypes before and after addition of VLDL or LDL to fasting plasma are shown in **B** , **D** , **F** , **H** , **J** (only three results for VLDL due to technical problems), and **L** , respectively. Lines combine fasting levels ( *open circles* ) with postprandial levels ( *closed circles* ) for each study participant, and for the additional study the levels before ( *open circles* ) and after addition of VLDL ( *blue closed circles* ) and LDL ( *red closed circles* ). Small bars to the left of the fasting and to the right of the postprandial results (or after addition of VLDL [ *blue* ] or LDL [ *red* ]) indicate the median of all results.

**Fig. 3:**
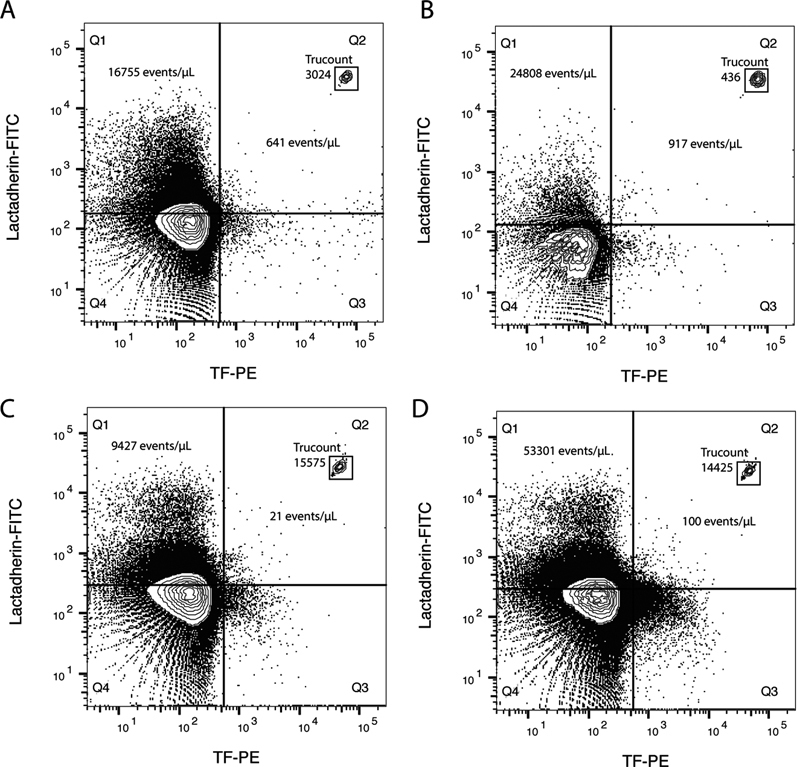
Representative flow cytometry scatter plots of ( **A** ) fasting and ( **B** ) postprandial PFP samples from one healthy donor and a sample before ( **C** ) and after ( **D** ) addition of VLDL. The number of TruCount beads and the number of TF− PS+ and TF+ PS+ particles are given in quadrant 1 (Q1) and 2 (Q2), respectively. Increased particle numbers lead to a higher coincident rate. Flow rates were, therefore, adjusted in postprandial samples with many events to maintain event rates below the maximal flow rate limit (<20,000 events/second) and thereby reduce coincident detection. Note: A lower flow rate reduces both the numbers of TruCount beads (the framed dots in quadrant 2) and PS+ events acquired in the postprandial scatter plot ( **B** ).

FC results on the fasting and postprandial samples are shown in Fig. 1 (concentrations of total and PS+ particles for each subject) and Fig. 2 (levels of antibody labeled subtypes). The concentration of total particles increased substantially in all subjects after food intake, the median rise being 14-fold (IQR, 5–30-fold). A less extensive, yet clearly statistically significant, postprandial increase in the concentration of PS+ particles and all studied subtypes of these, including TF+ PS+ particles, was observed. The relative increases from fasting to postprandial state are shown in Table 1 . Total TF+ particles (i.e., TF+ PS+ and TF+ PS− particles) were also detected and TF+ PS− particles constituted a considerable part of all TF+ particles and increased even more postprandially. Representative scatter plots of TF+ PS+ events in a fasting and a postprandial sample are shown in Fig. 3A , B .

**Table 1:** Changes in particle concentrations after food intake or in vitro lipoprotein addition

Study	Median (IQR) postprandial rise, (%) ( *n* = 20)	Median (range) rise after VLDL addition, (%) ( *n* = 4)	Median (range) rise after LDL addition, (%) ( *n* = 4)
Total particles	1,385*** (542–2,961)	365 (31–603)****	271 (23–434)****
Total PS+ particles	63*** (26–245)	379 (107–527)****	341 (56–683)****
TF+ PS+ particles	98*** (48–213)	132 (74–373)****	172 (26–336)****
TF+ PS− particles	449*** (171–1460)	272 (34–382)****	189 (12–263)****
Total TF+ particles	143*** (69–311)	266 (41–337)****	201 (15–251)****
CD41+ PS+ particles	103* (8–183)	18 (11–31)****	8 (1–15)****
CD146+ PS+ particles	49 **(11–121)	25 (6–157) a	36 (1–150)
CD62E+ PS+ particles	307*** (134–560)	25 (17–77)	61 (−1 to 101)

Abbreviations: IQR, interquartile range; LDL, low-density lipoprotein; PS+ , phosphatidylserine-positive; PS− , phosphatidylserine-negative; TF+ , tissue factor–positive; VLDL, very low-density lipoprotein.

Note: Effect of meal consumption or in vitro addition of VLDL or LDL on relative concentrations of total particles, total PS+ particles, TF+ PS+ particles, TF+ PS− particles, total TF+ particles, CD41+ PS+ particles, CD146+ PS+ particles, and CD62E+ PS+ particles in platelet-free plasma (PFP) measured by flow cytometry.

*p < 0.01; ** *p* < 0.001; *** *p* < 0.0001; **** *p* < 0.005 for the combined VLDL and LDL additions.

a Only three determinations.

Also shown in Table 1 are the relative increases after addition of lipoproteins in vitro. The absolute numbers are shown in Figs. 1 and 2 . This investigation was performed later than the previous investigation on the fasting/postprandial samples, and generally the numbers of particles were lower, but the data indicate that spiking PFP with either LDL or VLDL results in variably increased levels of measurable total particles, PS+ particles, and subgroups staining positive for TF, CD41, CD146, and CD62E, respectively. Since the measurements on samples with added VLDL or LDL showed an increased level of the particles measured in each subgroup after addition, it is highly significant for most of the subgroups: for each subgroup, *p* < 0.005, *n* = 8 ( Table 1 ). Representative scatter plots of TF+ PS+ events in a fasting sample before and after addition of VLDL are shown in Fig. 3C , D . In control samples, where PBS was spiked with LDL and VLDL, none of the antibody-labeled subtypes of PS+ particles appeared.

### EV Array

**Fig. 4:**
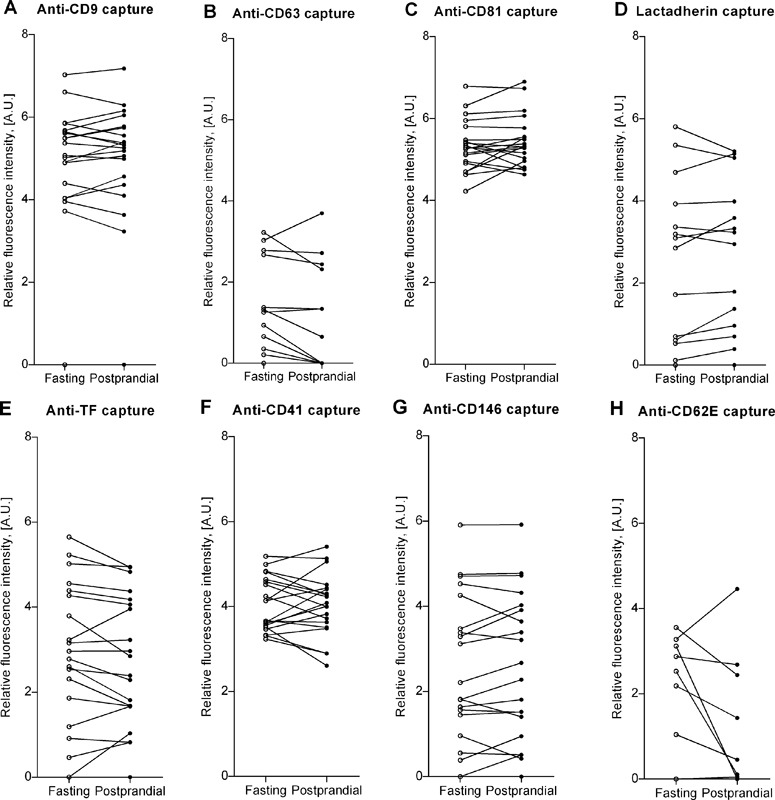
Fasting and postprandial content of small extracellular vesicles (EVs) in general (( **A** ) anti-CD9, ( **B** ) anti-CD63, and ( **C** ) anti-CD81 antibody capture), ( **D** ) phosphatidylserine-positive (PS+) small EVs (lactadherin capture), and small EV subtypes exposing specific antigens (( **E** ) anti–tissue factor (TF), ( **F** ) anti-CD41, ( **G** ) anti-CD146, and ( **H** ) anti-CD62E antibody capture) as semi-quantified by use of EV Array with an “exosome cocktail” consisting of fluorescently labeled antibodies against CD9, CD63, and CD81 as detecting agents. Lines combine fasting levels ( *open circles* ) with postprandial levels ( *closed circles* ) for each study participant. A.U., arbitrary units.

Fasting and postprandial fluorescence intensities measured with EV Array are shown in Fig. 4 . No significant differences between the fasting and postprandial samples were found. CD62E+ EVs were undetectable in the majority of the study participants, while the other investigated subgroups, including TF+ EVs and PS+ EVs (lactadherin capture), were detected in most subjects. We observed a substantial variation and markedly lower fluorescence intensities, including several below the detection limit, using anti-CD63 antibodies as compared with anti-CD9 and anti-CD81 antibodies. Results on the two last-mentioned exhibited consistency between fasting and postprandial results, showing a very low difference between the two measurements.

### PPL, TF Antigen, and TF Activity Analysis

**Fig. 5:**
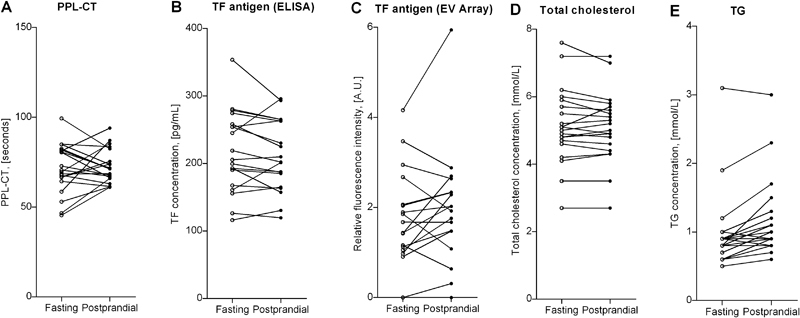
Fasting and postprandial results on ( **A** ) procoagulant phospholipid–dependent clotting time (PPL-CT), ( **B** ) tissue factor antigen (TF Ag) concentration as measured by ELISA, ( **C** ) TF Ag content as semi-quantified by EV Array using anti-TF antibodies as both capturing and detecting agent, ( **D** ) total cholesterol concentration, and ( **E** ) triglyceride (TG) concentration. Lines combine fasting levels ( *open circles* ) with postprandial levels ( *closed circles* ) for each study participant. A.U., arbitrary units.

**Fig. 6:**
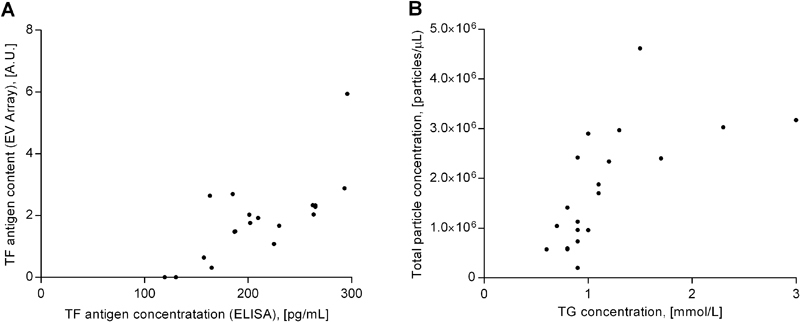
( **A** ) Correlation between tissue factor antigen (TF Ag) levels as measured by ELISA and EV Array in postprandial samples (Pearson's *r* = 0.73; *p* < 0.001). ( **B** ) Correlation between triglyceride (TG) concentration and total particle concentration measured by flow cytometry in postprandial samples (Spearman's *r _s_* = 0.79; *p* < 0.0001). A.U., arbitrary units.

Results on biological variation in PPL-CT and TF Ag levels as measured by TF Ag _ELISA_ and TF Ag _EV Array_ are given in Table 2 . No significant difference between fasting and postprandial samples was observed by pairwise comparison. Fasting and postprandial values for each subject are given in Fig. 5A – C . TF activity levels as measured by the chromogenic assay were below the limit of detection for all participants in the fasting as well as the postprandial state. A positive correlation was found between TF Ag _ELISA_ and TF Ag _EV Array_ in both postprandial ( Fig. 6A ) and fasting samples. Neither levels of TF+ PS+ particles (FC) nor TF+ EVs (EV Array) were significantly correlated with TF Ag levels in neither fasting nor postprandial samples. However, total TF+ particles (i.e., TF+ PS− and TF+ PS+ particles measured with FC) correlated moderately ( *r _s_* = 0.46; *p* = 0.04) with TF Ag _EV Array_ on the fasting samples and nonsignificantly with TF Ag _ELISA_ ( *r _s_* = 0.40; *p* = 0.09). PPL-CTs did not correlate with PS+ particle concentrations.

**Table 2:** Biological variation in procoagulant phospholipid–dependent clotting time (PPL-CT) and tissue factor antigen (TF Ag) levels in platelet-free plasma (PFP)

Study	Mean ± SD
PL-CT, a (s)	
Fasting state	71.3 ± 13.7
Postprandial state	72.6 ± 9.2
TF Ag _ELISA_ , b (pg/mL)	
Fasting state	217 ± 60
Postprandial state	211 ± 53
TF Ag _EV Array_ , a (AU)	
Fasting state	1.54 ± 1.16
Postprandial state	1.78 ± 1.35

Abbreviations: AU, arbitrary units.

a *n* = 20.

b For one subject, no ELISA results were obtained; hence, *n* = 19 for this method.

### Lipid Level Determination

Fasting and postprandial total cholesterol and TG concentrations for each subject are given in Fig. 5D , E . TG concentrations were significantly higher in the postprandial as compared with the fasting samples ( *p* < 0.001) with a mean increase of 0.2 mmol/L or 22%. No significant change in total cholesterol concentration was observed. A positive correlation was found between TG concentrations and total particle levels as measured by FC in postprandial ( Fig. 6B ) but not fasting samples.

## Discussion

In this study, we aimed to evaluate the effect of a shift from fasting to postprandial state on blood plasma levels of particles measured with FC and EVs measured with EV Array, especially of TF-bearing subtypes. Using FC, we observed significantly increased postprandial plasma levels of total particles, TF-bearing particles (PS− as well as PS+ particles), total PS+ particles, and subtypes exposing antigens indicating platelet and endothelial origin in the postprandial as compared with the fasting state. In vitro addition of lipoproteins to PFP also induced increased levels of all types of particles measured by FC in this study. No changes in total levels of EVs measured with EV Array, PPL-CT, or circulating TF Ag were observed postprandially and in no samples TF activity was above the limit of detection. Therefore, the seeming postprandial increases in MV concentrations (relying on the assumption that PS+ events represent MVs) measured with FC may, at least partially, be artifacts.

The postprandial increase in total particle concentration was expected and the magnitude of the increase agreed well with observations on the same study population counting particles by use of TRPS showing a median postprandial increase in particle concentrations by close to 1,400%. 12 Intestinal lipid absorption is followed by release of chylomicrons from enterocytes. 30 We expect these TG-rich lipoproteins, which have diameters between 75 and 1,200 nm, 30 to represent the main part of the increased number of particles measured after food intake. The positive correlation between the concentration of TG and total number of particles in the postprandial state supports this. The concentration of VLDLs, another type of TG-rich lipoprotein particles, is also markedly increased postprandially 31 32 but we would not expect single VLDL particles to be detected by FC as their diameter is appreciably below 100 nm except from a smaller fraction, named large VLDLs, with diameters up to 200 nm. 14 Interestingly, the amount of PS+ particles also increased considerably postprandially, but it is questionable to which degree the significant increases in PS+ particles and the measured subtypes really reflect changes in the MV population.

The nomenclature in the EV field is not consistent. 8 13 33 A distinction between MVs and exosomes, relying on their exposure of PS and specific tetraspanins, respectively, has been suggested but it is not possible in practice to differentiate between these two types of EVs. 18 Exosomes are actually not necessarily PS-free. 11 Although Lacroix et al advocated that identifying PS on particles using lactadherin seems preferable to distinguish true MV events from other particles in the same size range, 34 it has also been reported that a substantial part of circulating MVs do not expose measurable amounts of PS. 18 24 35 Moreover, Shi et al mentioned the possibility of lactadherin binding to lipoproteins that may themselves display PS. 20 With regard to exosomes, while tetraspanins considered to be exosome markers, i.e., CD9, CD63, and CD81, 36 are particularly abundant in the membranes of exosomes, they are also found in MVs. 22 Hence, we have designated particles detected by EV Array “small EVs” and particles detected with FC “PS+ particles” or “PS− particles.”

The combination of lactadherin for labeling particles exposing PS and an antibody for labeling cell type markers should theoretically help prevent lipoproteins from being mistaken for MVs. However, the fact that all the subtypes of PS+ particles that we measured in this study were more abundant in the postprandial state may indicate that FC counts of PS+ particles including any subgroup existing in PFP are in general increased as a result of interference from lipoproteins. Indeed, when we spiked plasma samples with VLDL or LDL, the results clearly indicated that the in vitro admixture of either type of lipoprotein prompted elevations of all examined subtypes of particles, although the magnitude of the rise varied substantially among the different subtypes, with the increase in CD41+ particles being least pronounced. Sódar et al recently demonstrated that LDLs do in fact adhere to MVs as well as to exosomes when mixed with them in vitro. 13 It could be speculated that LDLs adhering to vesicles smaller than those normally detectable by FC cause formation of fluorescently labeled particles large enough for light scatter–based detection. However, even without the occurrence of adhesion, lipoproteins and EVs simultaneously passing the optical system of the flow cytometer may conceivably be mistaken for a fluorescent particle within the size range detectable by FC, thus causing a variant of the phenomenon termed swarm detection. 37 Such mechanisms could explain the apparent increase in the antibody-labeled subtypes of PS+ particles postprandially. The TF+ PS− particles observed in our study may represent TF+ MVs that did not expose measurable amounts of PS or possibly TF+ exosomes attached to or “swarm detected” along with lipoproteins.

Our spiking study has some limitations. We did not add chylomicrons, which appear in high amounts in the true postprandial state. The sizes of LDLs and VLDLs in the added suspensions probably differ from their in vivo counterparts since previous studies have shown that the isolates contain particles with larger diameters than normally described for LDLs and VLDLs, probably due to aggregates of the lipoprotein particles. 38 FC of the samples spiked with VLDL or LDL was performed later and on other plasmas and other batches of antibodies, and consequently the numbers of particles were somewhat different from the first experiments. Nevertheless, the relative increases (experiments performed at the same time with the same type of antibodies) were clearly significant ( Table 1 ), and the results indicate that lipoproteins do interfere with the FC measurements. Also, we considered the possibility that the commercially purchased LDL and VLDL isolates may themselves contain some PS+ EV residuals, since co-purification of EVs and lipoproteins is a known phenomenon. 13 However, addition of LDLs and VLDLs in a solution containing PBS instead of PFP did not produce antibody-labeled events, indicating that this should not be a major source of error.

Although an artifactual effect on FC results of lipoproteins appearing postprandially is likely and although plasma content of smaller EVs measurable by EV Array did not change upon food intake, it is possible that part of the postprandial increase in antibody-labeled PS+ particles may actually be due to cellular MV release. Notably, among the PS+ particles, the CD62E+ fraction increased markedly more than the others, indicating a distinct effect on this subtype, which may reflect an increase in MVs from activated endothelial cells. Mechanistically, a rise in the number of CD62E+ PS+ MVs is plausible as increased plasma levels of TG have been shown to cause endothelial activation 39 40 and MVs can be released in response to cell activation. 41 Ferreira et al reported that postprandial hypertriglyceridemia induced elevated levels of endothelium-derived MVs both in the early (1 hour) postprandial phase and 3 hours after food intake. That study also relied on FC for MV detection, 42 thus being subject to the same type of possible interference from lipoproteins as the present study. Platelets can also be activated in the postprandial state. 43 44 Interestingly, Michelsen et al described elevated postprandial concentrations of platelet-derived MVs after a fat-rich meal using a non-FC-based immunometric method reportedly displaying a linear relation to FC results on platelet-derived MVs. 45 Our FC results on subtypes of CD41 + PS+ particles are consistent with these findings. Further elucidation of the potential interactions between MVs and lipoproteins and the relative contribution of either to the increase in cell type marker– and PS-bearing events measured by FC may be possible by addition of lipoprotein markers (e.g., anti-apolipoprotein B) or by detergent-based lysis of EVs as applied by Sódar et al. 13

The existence of circulating TF-bearing EVs in blood from healthy subjects has been described, 6 46 although it has also been stated that cells do not shed TF-bearing EVs under nonpathological conditions. 47 Since our various measurements of TF did not correlate very well, we clearly measured different populations of TF. However, consistency between fasting and postprandial EV Array results indicated that small TF+ EVs are not significantly influenced by food ingestion, whereas the reason for the postprandial increase of TF+ particles (PS+ and PS−) remains debatable. It would have been advantageous to measure TF activity but the results demonstrating levels below the limit of detection in all plasma samples are in accordance with the findings by others on healthy individuals. 48 49 Measurement of TF Ag may not solely measure active TF but the unaltered postprandial level indicates no major change of TF concentration postprandially. This is in accordance with the suggestion that the increase in TF+ particles measured with FC primarily reflects a higher number of small EVs adhering to or “swarming” alongside lipoproteins in formations that are large enough to be detected by FC rather than de novo release of TF+ MVs.

The finding that TF Ag levels are unaltered by food intake contrasts observations described by Motton et al. 48 However, postprandial blood samples in that study were drawn 3.5 and 6 hours following a standardized 40% fat meal that induced an increase in mean TG concentration by over 100% (3.5 hours postprandial), whereas the mean postprandial increase in TG concentration in our study confined itself to 22%. The postprandial samples in our study were drawn at a feasible time for blood sampling of patients in the morning after a normal breakfast. Sódar et al showed that the concentration of particles was almost maximal after 90 minutes. 13

Regarding PPL levels, our data demonstrated that although the amount of PS+ particles detectable by FC increased significantly in the postprandial state, this was not accompanied by a change in the coagulation state that can be monitored by PPL-CT. Tushuizen et al found that the total amount of PS in MV fractions was unaffected by food intake in healthy subjects, although PS+ MV concentration increased. 16 This corresponds well with the apprehension that a considerable part of the apparent postprandial increase in the concentration of PS+ particles may be an artifact resulting from increased lipoprotein interference.

## Conclusion

Food ingestion resulted in elevated plasma levels of PS+ particles, including subgroups exposing TF and other antigen markers measured by FC. While the observed postprandial increase may to some extent reflect elevated MV levels, the finding that in vitro addition of lipoproteins to fasting plasma had a similar effect suggests that it may at least partly be due to postprandially appearing lipoproteins. Thus, PS+ events that are also positive for TF or cell-specific antigen markers should not uncritically be interpreted as MVs, when performing FC-based investigations on MVs in blood.
